# Performance Analysis of Wireless Communications with Nonlinear Energy Harvesting under Hardware Impairment and *κ*-*μ* Shadowed Fading

**DOI:** 10.3390/s23073619

**Published:** 2023-03-30

**Authors:** Toi Le-Thanh, Khuong Ho-Van

**Affiliations:** 1 Ho Chi Minh City University of Technology (HCMUT), 268 Ly Thuong Kiet Street, District 10, Ho Chi Minh City 700000, Vietnam; lttoi.sdh19@hcmut.edu.vn; 2 Vietnam National University Ho Chi Minh City, Linh Trung Ward, Thu Duc District, Ho Chi Minh City 700000, Vietnam; 3 Ho Chi Minh City University of Food Industry, 140 Le Trong Tan Street, Tay Thanh Ward, Tan Phu District, Ho Chi Minh City 700000, Vietnam

**Keywords:** multiple antennas, energy-harvesting nonlinearity, hardware impairment, *κ*-*μ* shadowed fading, performance analysis

## Abstract

This paper improves energy efficiency and communications reliability for wireless transmission under κ-μ shadowed fading (i.e., integrating all channel impairments including path loss, shadowing, fading) and hardware impairment by employing a nonlinear energy harvester and multi-antenna power transmitter. To this end, this paper provides explicit formulas for outage probability. Numerous results corroborate these formulas and expose that energy-harvesting nonlinearity, hardware impairment, and channel conditions drastically deteriorate system performance. Notwithstanding, energy-harvesting nonlinearity influences system performance more severely than hardware impairment. In addition, desired system performance is accomplished flexibly and possibly by choosing a cluster of specifications. Remarkably, the proposed communications scheme obtains the optimal performance with the appropriate selection of the time-splitting factor.

## 1. Introduction

Next-generation wireless networks, e.g., 5G/6G, provide innumerable viable wireless applications for an enormous number of devices, yet induce critical pressures on telecommunications infrastructure, especially in current situations of energy deficiency, in supplying adequate energy for such devices [1,2,3,4]. Consequently, countermeasures ameliorating energy efficiency are becoming increasingly needed. One of the emerging countermeasures is to harvest energy available in radio frequency (RF) signals surrounding communications devices. Presently, 5G/6G devices deploy successfully cheap energy harvesting (EH) integrated circuits [5,6,7,8]. Nonetheless, a plurality of performance analyses pertinent to RF EH has featured EH to be linear for simplicity [9,10,11,12]. Practically, EH circuits are constituted by nonlinear elements, viz. capacitors, inductors, and diodes. As a result, featuring EH needs to account for the nonlinearity (NL) of circuit components. Until now, divergent nonlinear energy-harvesting (NLEH) paradigms have integrated such nonlinearity [13,14,15,16,17,18,19].

**Figure 1:**
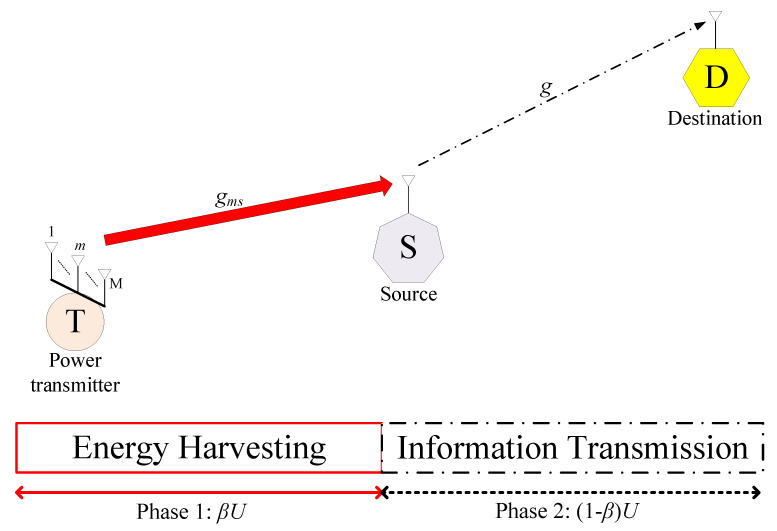
Wireless communications with energy harvesting.

Wireless communications with energy harvesting (WCwEH), e.g., Figure 1, enables a source S to transmit its information to a destination D. S harvests energy from a power transmitter T to accumulate power for its operation. Here, T can be television/radio broadcasting stations with high and stable transmission power. To further increase the quantity of harvested energy, multiple antennas should be deployed at T, which is the scenario in the current paper.

In practice, hardware impairment (HWi), which may originate from an imperfect design process (viz. in-quadrature-phase imbalances and phase noises) or imperfect hardware elements (viz., amplifier nonlinearities), is present in transceivers [20,21,22]. HWi plays a role as an interference source and therefore, in degrading system performance significantly [23]. Accordingly, it is mandatory to analyze and evaluate it elaborately in the system configuration process before implementation. Moreover, wireless communications are challenging due to propagation conditions, which include shadowing, fading, and path loss. These conditions happen concurrently and dramatically influence its performance. For WCwEH investigated in our work, propagation conditions also affect the quantity of harvested energy, eventually affecting communications reliability. For performance analysis practically, propagation conditions need to be featured appropriately to match field measurements. The κ-μ shadowed fading paradigm is extensively avouched in characterizing properly simultaneous influences of shadowing, fading, and path loss [24,25]. Remarkably, by varying a parameter group $\left(\kappa ,\mu ,\chi ,\delta \right)$ representing such a paradigm, divergent impairment degrees of shadowing, fading, and path loss can be set straightforwardly. The parameter δ indicates channel power including path loss, κ signifies the Rician-*K* element that represents the Line of Sight effect, χ stands for the shadowing effect, and μ denotes a sum of multi-path sets. As a result, this paradigm features a plurality of general-and-practical propagation conditions, including well-acknowledged fading distributions, viz., Rayleigh, one-sided Gaussian, Rice, Hoyt, Nakagami-*m*, etc. [25].

A few works [26,27,28,29] have studied wireless communications under concurrent consideration of energy-harvesting nonlinearity (EHNL) and hardware impairment. Nonetheless, these works have revealed several limitations. To be more specific, the authors in ref. [26] considered transmission from S to D under the aid of the reconfigurable intelligent surface operated as a relay. Although the authors in [26] analyzed rate-energy tradeoff, it investigated simple Rayleigh fading channels without accounting for shadowing, and missed the throughput (TP) and the outage probability (OP) analyses. Suffering from the same limitations as [26], the authors in [27] designed beamformers to obtain power efficiency and information security for direct transmission from S to multiple destinations. Nevertheless, ref. [27] studied Rician fading for the channel from T to S. Similar to [26,27] in which no analysis of the TP and the OP was presented, the authors in ref. [28] assessed the TP of the secondary network in the context of cognitive radio over Nakagami-*m* fading without taking shadowing into account. Recently, the authors in ref. [29] analyzed the TP and the OP of overlay networks over κ-μ shadowed fading which considers fading distributions (Rayleigh [26,27], Nakagami-*m* [28], Rician [27]) as special cases and accounts for shadowing and path loss. Nevertheless, only [27] considered multiple antennas at T for high energy efficiency and at S for beam-forming implementation, while [26,28,29] investigated the single antenna at T, therefore hardly improving energy-harvesting efficiency. Although multiple antennas at T increase considerably the quantity of harvested energy and therefore enhance the system performance, the performance analysis requires new statistics of harvested energy at S and therefore complicating analytical results. Briefly, the performance analysis for wireless communications through measures of the TP and the OP under simultaneous considerations of EHNL, HWi, multiple antennas at T, flexible-and-general κ-μ fading, shadowing, and path loss has been left open in the literature. This paper will solve this open problem to swiftly evaluate and maximize the reliability of the communication before practical implementation. More specifically, our contributions are presented as follows:

- Our work proposes WCwEH in Figure 1, wherein the power transmitter T employs an arbitrary quantity of antennas for ameliorating energy-harvesting efficiency, ultimately ameliorating communications reliability. To feature properly nonlinear circuit elements in energy harvesters, our work proposes the application of the extensively acknowledged NLEH paradigm in [18].
- To assess the reliability of the communication quickly, our work proposes the TP and the OP analyses for the recommended WCwEH under the consideration of EHNL, multi-antenna power transmitter, HWi, and divergent impairment degrees of shadowing, fading, and path loss in propagation conditions.
- Our work rate maximizes the reliability of communication in diverse realistic contexts. A plurality of results illustrates that EHNL, HWi, and propagation conditions drastically deteriorate system performance. Notwithstanding, EHNL influences the system performance more severely than HWi. In addition, the desired system performance is accomplished flexibly and possibly by choosing a cluster of specifications. Remarkably, the proposed transmission scheme obtains the optimal performance with the appropriate selection of the time-splitting factor.

Our work continues with Section 2, which presents the proposed WCwEH. Then, Section 3 analyzes the TP and the OP for the proposed WCwEH. Subsequently, Section 4 outlines the analyses for four extreme scenarios to facilitate quick performance comparison and highlights the impacts of EHNL, HWi, and multi-antenna deployment. Next, Section 5 provides simulated/analytical results in diverse practical settings. Finally, the paper is concluded in Section 6. Frequently used notations are tabulated in Table 1.

## 2. Wireless Communications with Energy Harvesting

### 2.1. System Model

Figure 1 demonstrates the basic system model of WCwEH with three devices (T, S, and D). Such a WCwEH may represent direct (uplink/downlink) transmission in mobile communications networks. S is supposed to be power-constrained and therefore it needs to harvest energy from T. T plays a role as a dedicated power transmitter, e.g., television and radio broadcasting stations. In the proposed WCwEH, T supplies energy for S’s operations in a time fraction β of a communications frame *U*, namely Phase 1, and S transmits its information transmission to D in the rest of *U*, namely Phase 2. To increase the quantity of harvested energy, eventually ameliorating communications reliability, T is assumed to be equipped with *M* antennas, which is feasible for T to be a high-transmission-power source. Indeed, S can harvest much more energy when T transfers energy through its higher number of antennas. Notwithstanding, since S and D may be mobile devices, the deployment of a single antenna on them is a better assumption. Furthermore, for realistic consideration, all devices are supposed to suffer from HWi.

### 2.2. Channel Model

We denote *g* as the channel power gain between S and D, and $g_{ms}$ as the channel power gain between S and the *m*th transmit antenna of T. We assume slow flat κ-μ shadowed fading channels. More specifically, a parameter set $\left(\mu ,\kappa ,\chi ,\delta_{tr}\right)$ specifies totally $g_{tr}$ with $g_{tr}\in \left\{g_{ms},g\right\}$ where (κ,μ,χ) were discussed in Section 1 and $\delta_{tr}=E\left\{g_{tr}\right\}$ with $\delta_{tr}\in \left\{\delta_{ms},\delta \right\}$ notates the corresponding channel power. In line with [24], the PDF and the CDF of $g_{tr}$ are respectively expressed to be

(1) $$f_{g_{tr}}\left(w\right)=\sum_{n=0}^{N}\frac{Q_{n}}{Y_{tr}^{\chi_{n}}\Gamma \left(\chi_{n}\right)}w^{\chi_{n}-1}e^{-\frac{w}{Y_{tr}}},$$

and

(2) $$F_{g_{tr}}\left(w\right)=1-\sum_{n=0}^{N}\sum_{i=0}^{\chi_{n}-1}\frac{Q_{n}}{Y_{tr}^{i}i!}w^{i}e^{-\frac{w}{Y_{tr}}},$$

where $Y_{tr}=\frac{\delta_{tr}\left(\chi +\kappa \mu \right)}{\mu \chi \left(\kappa +1\right)}$, $Q_{n}={\left(\frac{\kappa \mu }{\chi +\kappa \mu }\right)}^{N-n}{\left(\frac{\chi }{\chi +\kappa \mu }\right)}^{n}C_{n}^{N}$, $\chi_{n}=\chi -n$, N=χ−μ with μ≤χ. For compactness, μ≤χ and the same parameter set (κ, μ, χ) for all channels are supposed. In the context that μ>χ, we analyze similarly to μ≤χ by employing the corresponding symbols in [24] [Table I]. Furthermore, μ and χ are supposed to be integers, which have a slight impact in practicality as comprehended in [24]. Since shadowing and fading are already integrated into the κ-μ shadowed fading paradigm, we only need to embed path loss into this paradigm for wireless channels to be featured by simultaneous influences of fading, shadowing, and path loss. To this end, we model $\delta_{tr}$ as $\tau d_{tr}^{-\alpha }$ with α being path loss exponent, $d_{tr}$ being the corresponding transmitter-to-receiver distance, and τ being the fading power at the reference distance of 1 m (m) [13].

Wireless channels between S and the antennas of T are supposed to be independent and identically distributed (i.i.d). Accordingly, we write the subscript ms pertinent to channel parameters ($g_{ms}$, $Y_{ms}$, $\delta_{ms}$) shortly as *p* in ($g_{ms}$, $Y_{ms}$, $\delta_{ms}$) if not causing any confusion, namely $\varpi_{ms}=\varpi_{p}$, ∀m, ϖ={g,Y,δ}.

### 2.3. Signal Model

In Phase 1, T supplies energy for S over the multiple-input single-output channel, dramatically boosting the amount of harvested energy at S. Consequently, S harvests energy as $E=\eta \beta U\bar{P}\sum_{m=1}^{M}g_{ms}$ wherein 0<η<1 notates the energy conversion efficiency, $\bar{P}$ is the transmit power of each antenna of T, and $g_{ms}={\left|h_{ms}\right|}^{2}$ with $h_{ms}$ being the channel gain between S and the $m^{th}$ transmit antenna of T. Since the duration of Phase 2 is (1−β)U, the power for transmission in Phase 2 converted from *E* is $\frac{E}{(1-\beta )U}$. Conforming to the NLEH paradigm in [18], S transmits information in Phase 2 with the power as

(3) $$\begin{array}{cc} P & =\left\{\begin{array}{cc} \frac{\eta \beta \bar{P}}{1-\beta }\sum_{m=1}^{M}g_{ms} & ,\beta \bar{P}\sum_{m=1}^{M}g_{ms}\leq \;\iota \\ \frac{\eta \beta \iota }{1-\beta } & ,\beta \bar{P}\sum_{m=1}^{M}g_{ms}>\;\iota \end{array}\right.\\ \; & =\left\{\begin{array}{cc} GH & ,H\leq \;R\\ J & ,H>R \end{array}\right. \end{array}$$

where ι is the power saturation threshold, $G=\frac{\eta \beta \bar{P}}{1-\beta }$, $J=\frac{\eta \beta \iota }{1-\beta }$, $R=\frac{\iota }{\beta \bar{P}}$, and $H=\sum_{m=1}^{M}g_{ms}$.

It should be noted that Equation (3) reflects the characteristic of the NLEH. Indeed, the output power of the NLEH is GH, which is proportional linearly to its input power when the input power is below ι; otherwise, the output power of the NLEH is saturated at ι. Furthermore, we note that as ι is high (ι→∞), the NLEH reduces to the linear energy harvesting (LEH).

In Phase 2, S transmits its information *x* with transmit power *P* to D where $E\left\{{\left|x\right|}^{2}\right\}=1$. By accounting for HWi, D receives the signal to be [23]

(4) $$y=h\left(\sqrt{P}x+\upsilon \right)+\varsigma ,$$

wherein $\varsigma ∼CN\left(0,\sigma \right)$ is additive noise at D, *h* is the channel gain, $\upsilon ∼CN\left(0,\rho P\right)$ is HWi at S and D where ρ is the total HWi at S and D.

The signal-to-interference plus noise ratio (SINR) for D to restore *x* from Equation (4) is

(5) $$\Lambda =\frac{gP}{\rho gP+\sigma }.$$

where $g={\left|h\right|}^{2}$ is the channel power gain.

It is drawn from Equation (5) that HWi plays a role as an interference source generating the quantity of interference as ρgP. This interference causes performance degradation in comparison to hardware perfection.

## 3. Performance Analysis of WCwEH

The OP of WCwEH is first analyzed in this part. The OP refers to the probability that D decodes unsuccessfully *x*, i.e., the achieved channel capacity is below the target transmission rate $R_{0}$. Subsequently, the OP analysis is extended to achieve the TP analysis. These proposed analyses facilitate the quick OP/TP evaluation without time-consuming simulations.

### 3.1. Exact Analysis

The communications reliability is represented by the outage probability at D. Therefore, the lower the OP at D, the higher the reliability of the communication. The OP at D is expressed to be

(6) $$\begin{array}{cc} O & =P\left\{\Lambda <\Lambda_{0}\right\}\\ \; & =P\left\{\frac{gP}{\rho gP+\sigma }<\Lambda_{0}\right\}\\ \; & =\left\{\begin{array}{cc} \tilde{O} & ,1>\Lambda_{0}\rho \\ 1 & ,1\leq \Lambda_{0}\rho \end{array}\right. \end{array}$$

where (Since the duration of Phase 2 is (1−β)U, the channel capacity corresponding to the SINR Λ in Phase 2 is $\left(1-\beta \right)\log_{2}\left(1+\Lambda \right)$. The outage event happens if this capacity is below $R_{0}$ or the SINR Λ is lower than $\Lambda_{0}$). w=gP, $\Lambda_{0}=2^{R_{0}/\left(1-\beta \right)}-1$, and

(7) $$\begin{array}{cc} \tilde{O} & =P\left\{w<\frac{\Lambda_{0}\sigma }{1-\Lambda_{0}\rho }\right\}\\ \; & =F_{w}\left(\frac{\Lambda_{0}\sigma }{1-\Lambda_{0}\rho }\right). \end{array}$$

It is drawn from Equation (6) that since $\Lambda_{0}=2^{R_{0}/\left(1-\beta \right)}-1$, choosing the target transmission rate $R_{0}$, the time-splitting factor β, and the HWi ρ may induce $\Lambda_{0}\rho \geq 1$, causing O to be 1 or leading WCwEH to be in a complete outage. However, this complete outage event can be prevented by selecting properly {$R_{0}$, β, ρ} such that $\Lambda_{0}\rho <1$. This insight can be drawn from the condition $\Lambda_{0}\rho <1$ to avoid the complete outage event as follows. It is seen that $\Lambda_{0}\rho <1$ is equivalent to $R_{0}<\left(1-\beta \right)\log_{2}\left(1+\rho^{-1}\right)$, which means that for WCwEH to prevent the complete outage, the target transmission rate must be upper-bounded properly. The upper bound on $R_{0}$ depends on the parameter of energy-harvesting β and the HWi ρ. The higher the β (or ρ), the lower target transmission rate the WCwEH achieves. The higher β means more time for energy harvesting while less time for signal transmission. Similarly, the higher ρ means the HWi is more severe. Furthermore, O depends on the parameter set $(R_{0}$, β, $\bar{P}$, *M*, ι, η, ρ), which means that S can achieve the desired performance by properly setting this set.

To complete the OP analysis, we must derive the CDF of *w*, $F_{w}\left(z\right)$, in Equation (7), which is addressed as

(8) $$F_{w}\left(z\right)=P\left\{gP\leq z\right\}.$$

Invoking *P* in Equation (3), the CDF of *w* is further represented to be

(9) $$\begin{array}{cc} F_{w}\left(z\right) & =P\left\{g\leq \frac{z}{GH},H\leq \;R\right\}+P\left\{g\leq \frac{z}{J},H>R\right\}\\ \; & =\int_{0}^{R}F_{g}\left(\frac{z}{Gx}\right)f_{H}\left(x\right)dx+{\bar{F}}_{H}\left(R\right)F_{g}\left(\frac{z}{J}\right). \end{array}$$

The integral in Equation (9) is approximated exactly by employing the Gaussian–Chebyshev quadrature in [31] as

(10) $$F_{w}\left(z\right)=\frac{\pi R}{2I}\sum_{i=1}^{I}\sqrt{1-\varepsilon_{i}^{2}}f_{H}\left(\nu_{i}\right)F_{g}\left(\frac{z}{\nu_{i}G}\right)+{\bar{F}}_{H}\left(R\right)F_{g}\left(\frac{z}{J}\right),$$

where $\epsilon_{i}=\cos\left(\frac{2i-1}{2I}\pi \right)$, $\nu_{i}=R\left(\varepsilon_{i}+1\right)/2$, and *I* stands for the accuracy–complexity tradeoff of the Gaussian–Chebyshev quadrature. In Section 5, we demonstrate the results with I=200 that guarantees very high preciseness.

To complete the derivation of Equation (10), we must derive the PDF and the CCDF of $H=\sum_{m=1}^{M}g_{ms}$. To this end, it is noted that the MGF of the sum of *M* i.i.d random variables is a product of *M* individual MGFs. Applying this note, one obtains the MGF of *H* to be

(11) $$\begin{array}{cc} \Psi_{H}\left(v\right) & =\prod_{m=1}^{M}\Psi_{g_{ms}}\left(v\right)\\ \; & ={\left[\Psi_{g_{p}}\left(v\right)\right]}^{M}. \end{array}$$

In Equation (11), the first equality originated from the statistical independence of *M* random variables while the second equality is comprehended from their identical distributions. Furthermore, each MGF is represented as

(12) $$\begin{array}{cc} \Psi_{g_{p}}\left(v\right) & =\int_{0}^{\infty }f_{g_{p}}\left(y\right)e^{vy}dy\\ \; & =\int_{0}^{\infty }\left(\sum_{n=0}^{N}\frac{Q_{n}}{Y_{p}^{\chi_{n}}\Gamma \left(\chi_{n}\right)}y^{\chi_{n}-1}e^{-\frac{y}{Y_{p}}}\right)e^{vy}dy\\ \; & =\sum_{n=0}^{N}\frac{Q_{n}}{Y_{p}^{\chi_{n}}\Gamma \left(\chi_{n}\right)}\int_{0}^{\infty }y^{\chi_{n}-1}e^{-\left(\frac{1}{Y_{p}}-v\right)y}dy\\ \; & =\sum_{n=0}^{N}Q_{n}{\left(1-Y_{p}v\right)}^{-\chi_{n}}. \end{array}$$

By substituting (12) into (11) and applying the multinomial expansion, one obtains the MGF of *H* as

(13) $$\begin{array}{cc} \Psi_{H}\left(v\right) & ={\left[\sum_{n=0}^{N}Q_{n}{\left(1-Y_{p}v\right)}^{-\chi_{n}}\right]}^{M}\\ \; & =\sum_{\sum_{n=0}^{N}b_{n}=M}\frac{M!}{\prod_{n=0}^{N}b_{n}!}\prod_{n=0}^{N}{\left[Q_{n}{\left(1-Y_{p}v\right)}^{-\chi_{n}}\right]}^{b_{n}}\\ \; & =\sum_{\sum_{n=0}^{N}b_{n}=M}M!\left\{\prod_{n=0}^{N}\frac{{\left(Q_{n}\right)}^{b_{n}}}{b_{n}!}\right\}{\left(1-Y_{p}v\right)}^{-\vartheta }, \end{array}$$

where $\vartheta =\sum_{n=0}^{N}b_{n}\chi_{n}$.

Using the PDF-MGF mapping in [32], one infers the PDF of *H* to be

(14) $$f_{H}\left(z\right)=\sum_{\sum_{n=0}^{N}b_{n}=M}\left(\prod_{n=0}^{N}\frac{{\left(Q_{n}\right)}^{b_{n}}}{b_{n}!}\right)\frac{M!z^{\vartheta -1}}{Y_{p}^{\vartheta }\Gamma \left(\vartheta \right)}e^{-\frac{z}{Y_{p}}}.$$

Subsequently, the CCDF of *H* is expressed from its definition as ${\bar{F}}_{H}\left(z\right)=P\left\{H\geq z\right\}=\int_{z}^{\infty }f_{H}\left(r\right)dr$. Plugging (14) into ${\bar{F}}_{H}\left(z\right)$ yields

(15) $${\bar{F}}_{H}\left(z\right)=\sum_{\sum_{n=0}^{N}b_{n}=M}\frac{M!}{Y_{p}^{\vartheta }\Gamma \left(\vartheta \right)}\left(\prod_{n=0}^{N}\frac{{\left(Q_{n}\right)}^{b_{n}}}{b_{n}!}\right)\int_{z}^{\infty }r^{\vartheta -1}e^{-\frac{r}{Y_{p}}}dr.$$

With the aid of ([30], Equation (3.351.2)), one reduces (15) to

(16) $${\bar{F}}_{H}\left(z\right)=\sum_{\sum_{n=0}^{N}b_{n}=M}\frac{M!}{\Gamma \left(\vartheta \right)}\Gamma \left(\vartheta ,\frac{z}{Y_{p}}\right)\prod_{n=0}^{N}\frac{{\left(Q_{n}\right)}^{b_{n}}}{b_{n}!}.$$

### 3.2. Asymptotic Analysis

This subsection analyzes the upper bound in the performance of WCwEH in the range of high transmit power (i.e., $\bar{P}\to \infty$). It should be noted that the energy scavenger becomes saturated completely when $\bar{P}\to \infty$. In other words, $\bar{P}\to J$ when $\bar{P}\to \infty$. Therefore, the CDF of w=gJ reduces to $F_{w}\left(z\right)=F_{g}\left(\frac{z}{J}\right)$ as $\bar{P}\to \infty$. As a result, the OP of WCwEH is derived as

(17) $$O^{\infty }=\left\{\begin{array}{cc} F_{g}\left(\frac{\Lambda_{0}\sigma }{\left[1-\Lambda_{0}\rho \right]J}\right) & ,1\geq \Lambda_{0}\rho \\ 1 & ,1<\Lambda_{0}\rho \end{array}\right.$$

### 3.3. Throughput

For WCwEH with delay-limited transmission, the throughput is effortlessly inferred from the OP analysis to be

(18) $$T=\left(1-\beta \right)R_{0}\left(1-O\right).$$

Relied on (18), one observes that the throughput of WCwEH is also jointly affected by the multi-parameter set $(R_{0}$, β, $\bar{P}$, *M*, ι, η, ρ) because this set determines completely O. As a result, a desired throughput is attained by setting these parameters flexibly and properly based on their predetermined value ranges.

## 4. Extreme Scenarios

This section considers four extreme scenarios for WCwEH: (1) hardware perfection (HWp) and nonlinear energy harvesting; (2) hardware impairment (HWi) and linear energy harvesting; (3) hardware perfection and linear energy harvesting; (4) single-antenna power transmitter M=1. These scenarios facilitate quick comparisons with the analytical results in Section 3 to highlight joint/individual influences of HWi/HWp, NLEH/LEH, and the number of antennas at T. It is worth noting that the performance analyses for these extreme scenarios have not yet been reported, and constitute future contributions of our work.

### 4.1. Hardware Perfection and Nonlinear Energy Harvesting (HWpNLEH)

Let $O_{1}$ denote the OP in the scenario of HWpNLEH (viz., ρ=0 and ι<∞). By setting ρ=0 in the expressions in Section 3, one obtains $O_{1}$ as

(19) $$O_{1}=F_{w}\left(\Lambda_{0}\sigma \right).$$

The result in (19) reveals that the multi-parameter set $(R_{0}$, β, $\bar{P}$, *M*, ι, η) completely determines $O_{1}$. Moreover, (19) helps study the individual influence of HWi by comparing it with the analytical result from Section 3.

### 4.2. Hardware Impairment and Linear Energy Harvesting (HWiLEH)

The outage performance of WCwEH in the scenario of HWiLEH (viz., ρ>0 and ι→∞) is analyzed in the following for prompt comparison with its nonlinearity counterpart in Section 3, eventually highlighting the individual effect of EHNL on the reliability of the communication. It is noted that the scenario of HWiLEH means P→GH and therefore, the CDF of *w* becomes

(20) $$\begin{array}{cc} {\tilde{F}}_{w}\left(v\right) & =\mathrm{Pr}\left\{g\leq \frac{v}{GH}\right\}\\ \; & =\int_{0}^{\infty }F_{g}\left(\frac{v}{Gz}\right)f_{H}\left(z\right)dz. \end{array}$$

Plugging $F_{g}\left(\cdot \right)$ in (2) and $f_{H}\left(\cdot \right)$ in (14) into (20) after elaborate manipulations, one simplifies ${\tilde{F}}_{w}\left(v\right)$ as

(21) $${\tilde{F}}_{w}\left(v\right)=1-\sum_{n=0}^{N}\sum_{i=0}^{\chi_{n}-1}\sum_{\sum_{n=0}^{N}b_{n}=M}\frac{Q_{n}}{i!}{\left(\frac{v}{GY}\right)}^{i}\left(\prod_{n=0}^{N}\frac{{\left(Q_{n}\right)}^{b_{n}}}{b_{n}!}\right)\frac{M!}{Y_{p}^{\vartheta }\Gamma \left(\vartheta \right)}\int_{0}^{\infty }z^{\vartheta -i-1}e^{-\frac{v}{YGz}-\frac{z}{Y_{p}}}dz.$$

With the aid of [30] [Equation (3.471.9)], the integral in (21) can be solved, and therefore

(22) $${\tilde{F}}_{w}\left(v\right)=1-\sum_{n=0}^{N}\sum_{i=0}^{\chi_{n}-1}\sum_{\sum_{n=0}^{N}b_{n}=M}\left(\prod_{n=0}^{N}\frac{{\left(Q_{n}\right)}^{b_{n}}}{b_{n}!}\right)\frac{2Q_{n}M!}{i!\Gamma \left(\vartheta \right)}{\left(\frac{v}{YGY_{p}}\right)}^{\frac{\vartheta +i}{2}}K_{\vartheta -i}\left(2\sqrt{\frac{v}{YGY_{p}}}\right).$$

Following the procedure in Section 3.1, one derives the outage performance of WCwEH in the scenario of HWiLEH as

(23) $$O_{2}=\left\{\begin{array}{cc} {\tilde{F}}_{w}\left(\frac{\Lambda_{0}\sigma }{1-\Lambda_{0}\rho }\right) & ,1>\Lambda_{0}\rho \\ 1 & ,1\leq \Lambda_{0}\rho \end{array}\right.$$

The result in (23) is convenient in demonstrating the individual influence of EHNL on the reliability of the communications by comparing it with (6).

### 4.3. Hardware Perfection and Linear Energy Harvesting (HWpLEH)

Integrating the results in Section 4.1 and Section 4.2, one obtains the outage performance of WCwEH in the scenario of HWpLEH as

(24) $$O_{3}={\tilde{F}}_{w}\left(\Lambda_{0}\sigma \right).$$

The result in (24) is convenient in exposing the joint influences of EHNL and HWi on the system performance by comparing it with (6).

### 4.4. Hardware Impairment and Nonlinear Energy Harvesting with M=1 (HWiNLEHw1)

In the scenario of HWiNLEHw1, *H* becomes $g_{p}$ and therefore, the CDF of *w* reduces to

(25) $${\hat{F}}_{w}\left(v\right)=\frac{\pi R}{2I}\sum_{i=1}^{I}\sqrt{1-\varepsilon_{i}^{2}}F_{g}\left(\frac{v}{\nu_{i}G}\right)f_{g_{p}}\left(\nu_{i}\right)+F_{g}\left(\frac{v}{J}\right){\bar{F}}_{g_{p}}\left(R\right).$$

Consequently, the outage performance of WCwEH in the scenario of HWiNLEHw1 as

(26) $$O_{4}=\left\{\begin{array}{cc} {\hat{F}}_{w}\left(\frac{\Lambda_{0}\sigma }{1-\Lambda_{0}\rho }\right) & ,1>\Lambda_{0}\rho \\ 1 & ,1\leq \Lambda_{0}\rho \end{array}\right.$$

It should be noted that even though [29] studied the performance of overlay networks over κ−μ shadowed fading under concurrent effects of EHNL and HWi, it did not present the performance analysis for WCwEH with M=1. Therefore, the result in (26) is still novel and convenient in unveiling the individual impact of the number of antennas at T on the system performance by comparing it directly with (6).

## 5. Illustrative Results

This part presents a plurality of analytical/simulated results to evaluate the OP of WCwEH in numerous parameters. In the following, analytical results (Ana.) are produced by computing the analytical formulas derived in Section 3 and Section 4. Moreover, simulated results (Sim.) are generated by Monte Carlo simulations for comparison between analytical and simulated results to corroborate the analytical formulas. Due to linear mapping between the OP and the TP as mentioned in (18), the TP is computed directly from the OP. Consequently, this part focuses solely on the OP. For illustration, devices are located arbitrarily in a two-dimension plane and parameters are selected as in Table 2 unless otherwise addressed. The following figures indicate that (1) the analysis matches exactly the simulation, verifying the preciseness of the derived formulas in Section 3 and Section 4; (2) increasing *M* improves considerably the communications reliability, which is due to the increase in harvested energy, as expected.

**Figure 2:**
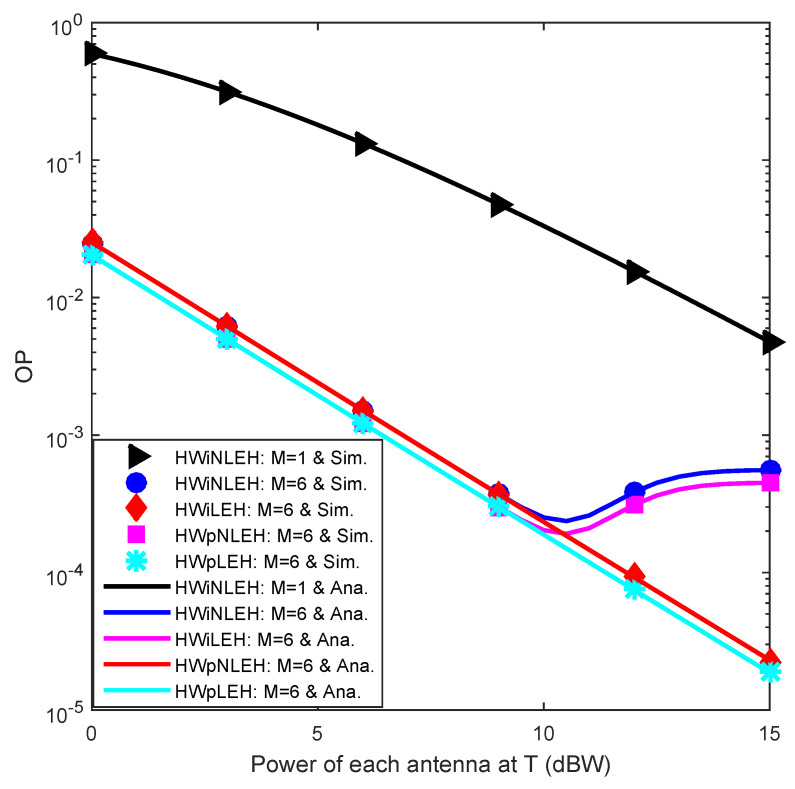
OP versus $\bar{P}$.

Figure 2 illustrates the OP against $\bar{P}$, which reveals the considerable reliability improvement (viz., smaller OP) with increasing $\bar{P}$ for five scenarios: (1) hardware impairment and nonlinear energy harvesting (HWiNLEH) with M=1; (2) hardware impairment and nonlinear energy harvesting (HWiNLEH) with M=6; (3) hardware impairment and linear energy harvesting (HWiLEH) with M=6; (4) hardware perfection and nonlinear energy harvesting (HWpNLEH) with M=6; (5) hardware perfection and linear energy harvesting (HWpLEH) with M=6. This is reasonable owing to increasing harvested energy. Additionally, HWi slightly degrades the outage performance (i.e., the OP in the scenario of HWiNLEH/HWiLEH is slightly higher than that in the scenario of HWpNLEH/HWpLEH). Moreover, the effect of EHNL is neglected at low $\bar{P}$ (i.e., the OP in the scenario of HWiNLEH/HWpNLEH is identical to that in the scenario of HWiLEH/HWpLEH for $\bar{P}<9.5$ dBW). This is because the nonlinear energy harvesting becomes the linear energy harvesting at low $\bar{P}$. However, at high $\bar{P}$ where the nonlinear energy harvester is saturated, EHNL significantly mitigates the outage performance (i.e., the OP in the scenario of HWiNLEH/HWpNLEH is greatly higher than that in the scenario of HWiLEH/HWpLEH for $\bar{P}>9.5$ dBW) and the performance gap between LEH and NLEH continues to enlarge with increasing $\bar{P}$, showing the detrimental impact of EHNL. Therefore, EHNL impacts the performance of the WCwEH more significantly than HWi.

**Figure 3:**
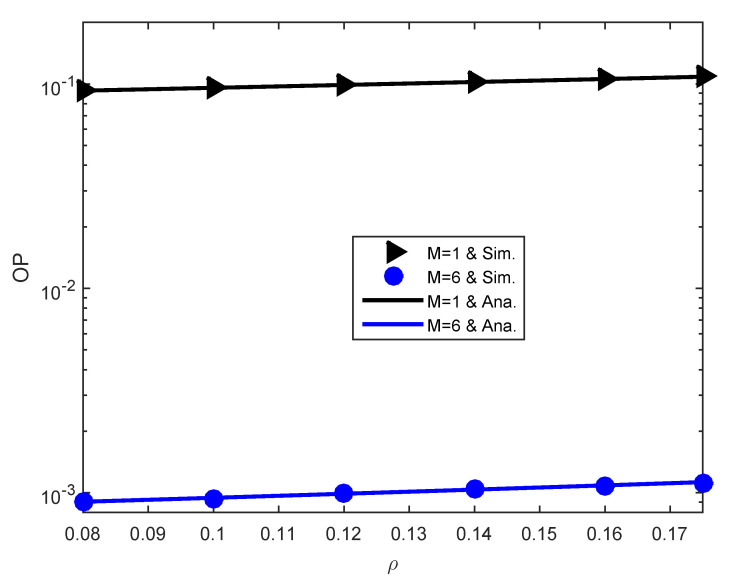
OP versus the HWi degree ρ.

Figure 3 unveils the influence of the HWi on the outage performance. As per 3GPP LTE [33], the standard range of ρ is from 0.08 to 0.175 where the physical meaning of ρ is error vector magnitude. One notices from Figure 3 that the reliability of the communication is slightly deteriorated by HWi with accreting ρ, which is reasonable because of the increase in the interference caused by HWi. This figure again emphasizes that EHNL impacts the performance of WCwEH more significantly than HWi.

**Figure 4:**
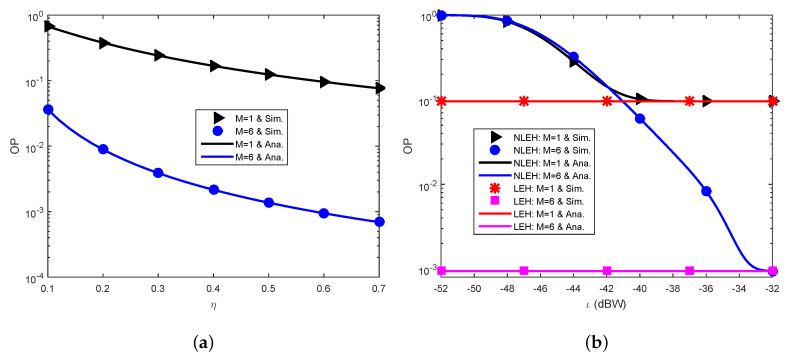
Parameters pertinent to harvested energy. (**a**) OP versus η. (**b**) OP versus ι.

Figure 4 demonstrates the OP against the energy conversion efficiency η (Figure 4a) and the power saturation threshold ι (Figure 4b) at the energy harvester of S. It is expected that increasing η and ι makes S harvest more energy, therefore decreasing the OP. Figure 4 demonstrates accurately this expectation wherein the reliability of the communication is dramatically ameliorated with increasing η and ι. Additionally, S incurs outage performance saturation at large ι at which the NLEH matches the LEH, as expected. Furthermore, S experiences a complete outage for small ι, as predicted.

**Figure 5:**
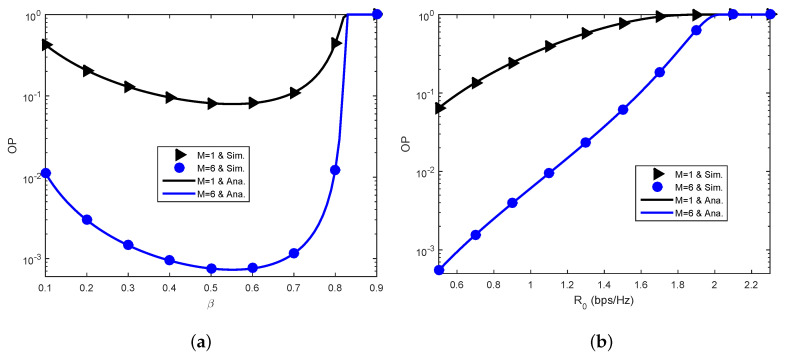
Effects of parameters $\left(\beta ,R_{0}\right)$. (**a**) OP versus β. (**b**) OP versus $R_{0}$.

Figure 5 exposes the OP against the time-splitting factor β (Figure 5a) and the target transmission rate $R_{0}$ (Figure 5b). Figure 5a shows that β can be optimized to attain optimal communications reliability. The optimal value of β is comprehended to poise the duration for energy harvesting (Phase 1) and the duration for transmission (Phase 2). Moreover, Figure 5a shows that S incurs a complete outage for β≥0.827, which is reasonable as analyzed in Section 3 where the OP is 1 when $\Lambda_{0}\rho \geq 1$. Given $R_{0}=0.6$ bps/Hz, ρ=0.1 and $\Lambda_{0}=2^{R_{0}/\left(1-\beta \right)}-1$, it is obvious that $\Lambda_{0}\rho \geq 1$ is equivalent to β≥0.827. Additionally, Figure 5b demonstrates the considerable performance degradation with increasing $R_{0}$, as predicted. Furthermore, S also incurs a complete outage for $R_{0}\geq 2.076$ bps/Hz, which coincides with the analysis in Section 3 where the OP is 1 when $\Lambda_{0}\rho \geq 1$. Given β=0.4, ρ=0.1 and $\Lambda_{0}=2^{R_{0}/\left(1-\beta \right)}-1$, it is obvious that $\Lambda_{0}\rho \geq 1$ is equivalent to $R_{0}\geq 2.076$ bps/Hz.

**Figure 6:**
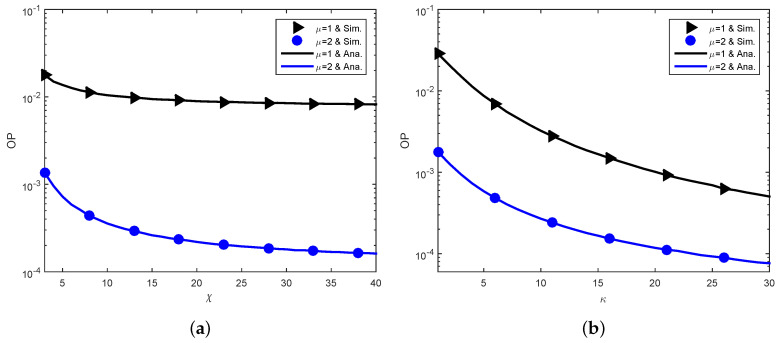
Shadowed fading parameters (μ,χ,κ). (**a**) OP versus χ. (**b**) OP versus κ.

Figure 6 demonstrates the impact of shadowed fading parameters (μ,χ,κ) on the outage performance. It should be noted that this paper considers integer values of (χ,μ) and the scenario of χ≥μ. It is observed from Figure 6 that the reliability of the communication is considerably ameliorated with increasing (κ,χ,μ), as anticipated.

## 6. Conclusions

Our work presented the outage performance analysis for WCwEH under practical conditions including hardware impairment, multi-antenna configuration, path loss, fading, shadowing, and nonlinear energy harvesting. Various results show that these conditions considerably affect the outage performance. Nonetheless, energy-harvesting nonlinearity influences the reliability of communication more severely than hardware impairment. Moreover, the system performance can be maximized with the appropriate selection of the time-splitting coefficient. Furthermore, the proper selection of the target transmission rate, the hardware impairment level, and the time-splitting factor can prevent the system from a complete outage.

## Figures and Tables

**Table 1:** Frequentlyused notations.

Notation	Interpretation
$CN\left(0,c\right)$	complex Gaussian random variable with mean 0 and variance *c*
Γ(·,·)	Incomplete upper Gamma function
$C_{m}^{n}=\frac{n!}{m!\left(n-m\right)!}$	Binomial coefficient
${\bar{F}}_{N}\left(\cdot \right)$	complementary cumulative distribution function (CCDF) of *N*
$\Psi_{N}\left(\cdot \right)$	Moment Generating Function (MGF) of *N*
$F_{N}\left(\cdot \right)$	cumulative distribution function (CDF) of *N*
P{·}	Probability operator
E{·}	Expectation operator
Γ(·)	Complete Gamma function
$K_{i}\left(\cdot \right)$	Modified Bessel function [30]
$f_{N}\left(\cdot \right)$	probability density function (PDF) of *N*

**Table 2:** Selected parameters unless otherwise addressed.

Parameter	Value
Location of T	(0,0) m
Location of S	(5,5) m
Location of D	(50,0) m
Noise power	σ=−90 dBm
Path loss exponent	α=3
Fading power at the reference distance of 1 m	$\tau =10^{-2}$
Shadowed fading parameters (κ,χ,μ)	(3, 4, 2)
HWi	ρ=0.1
Power saturation threshold	ι=0 dBm
Energy conversion efficiency	η=0.6
Time-splitting factor	β=0.4
Transmit power of each antenna of T	$\bar{P}=7$ dBW
Target transmission rate	$R_{0}=0.6$ bps/Hz
Quantity of antennas at T	M=6

## Data Availability

Data is contained within the article.
